# Macular and Optic Disc Perfusion Changes After Silicone Oil Removal Using Optical Coherence Tomography Angiography: A Prospective Study

**DOI:** 10.7759/cureus.56442

**Published:** 2024-03-19

**Authors:** Christina Karakosta, Vassilios S Verykios, Georgios Feretzakis, Christina Kourentis

**Affiliations:** 1 Ophthalmology Department, National and Kapodistrian University of Athens School of Medicine, Athens, GRC; 2 School of Science and Technology, Hellenic Open University, Patras, GRC; 3 First Ophthalmology Department, Ophthalmiatreio Eye Hospital of Athens, Athens, GRC

**Keywords:** optic disk, macula, retinal detachment surgery, silicone oil removal, oct angiography

## Abstract

Aim: The aim of this study was to prospectively evaluate the changes in macular and optic disc microvascular structures in patients who underwent silicone oil (SO) removal.

Materials and methods: A total of 28 patients scheduled for unilateral SO removal were included in the study. Their fellow eyes served as controls. Optical coherence tomography angiography (OCTA) of the retina (6.0 mm) and disc (4.5 mm) was performed one day before SO removal, and then at 1 week and 1, 3, 6, and 12 months postoperatively. All analyses were conducted using the R programming language, with a p-value <0.05 considered statistically significant.

Results: After silicone oil removal, statistically significant changes were observed in the flow in the outer retina and radial peripapillary capillary (RPC) density for small and all vessels inside the disc. Statistically significant differences between the intervention and control groups were noted in vessel density in both the superficial and deep capillary plexuses and RPC density for small and all vessels.

Conclusion: Changes in macular vessel density and radial peripapillary capillary density were observed after SO removal. The latter changes appear to improve after the first postoperative month and continue until the first postoperative year. Notably, these changes were significant between the first postoperative week and 6 and 12 postoperative months (p = 0.0263 and p = 0.021, respectively). Best corrected visual acuity (BCVA) is likely associated with these parameters, indicating that improvement may be observed even one year following SO removal.

## Introduction

Tamponade agents are used in retinal detachment (RD) repair and include silicone oil (SO) and gases [1]. The most common gas tamponades used are air, sulfur hexafluoride (SF6), perfluoropropane (C3F8), and perfluoroethane (C2F6). Unlike gas tamponades, SO does not resorb spontaneously and must be surgically removed [1].

The most common indications for SO tamponade include rhegmatogenous RD with proliferative vitreoretinopathy (PVR) or with a high risk of developing PVR, giant tears, severe diabetic retinopathy, uveitis, particularly viral retinitis, ocular trauma, and endophthalmitis [2,3]. Additionally, SO is preferred in patients who are unable to maintain the required head position or who need to travel at high altitudes [2]. However, SO tamponade has been linked to certain complications, among which the most common are cataract formation, glaucoma, and keratopathy [2,4]. For this reason, most surgeons prefer to remove SO within an interval of no longer than 3-6 months [3]. Nevertheless, unexplained visual loss following SO removal has been reported, and, even though certain hypotheses such as phototoxicity, the vitreous potassium sink theory, and alterations in retinal blood perfusion have been developed, the phenomenon is still not clearly understood [5].

Optical coherence tomography angiography (OCTA) is a non-invasive imaging technique that provides structural and blood flow information about both retinal vasculature and optic disc perfusion [6,7].

The aim of this study was to prospectively evaluate macular and optic disc microvascular structure changes in patients who underwent SO removal. To the authors' knowledge, this is the first study that prospectively investigated macular and optic disc changes occurring up to 1 year following SO removal.

## Materials and methods

Participants

This prospective study was conducted in the Ophthalmology Department of a tertiary care facility. The study's protocol was approved by the Institutional Ethics Committee at Ophthalmiatreio Eye Hospital of Athens (9398/20-02-2022), adhering to the Declaration of Helsinki principles. Informed consent was obtained from all patients participating in the study.

Patients with unilateral rhegmatogenous RD who underwent surgical repair with SO tamponade between March 2022 and September 2022 were included in the study. SO was removed within three to six months for all patients, with the observation period extending up to 12 months.

Exclusion criteria were glaucoma, coexisting ocular conditions that could potentially impair visual function (such as diabetic retinopathy/uveitis with macular edema, macular hole, or age-related macular degeneration), corneal opacities that might affect OCTA imaging quality, a history of trauma, and second surgery due to failure of retinal reattachment. The healthy fellow eyes served as controls, dividing participants into two groups: the intervention group (eyes that underwent SO removal) and the control group (fellow unoperated eyes).

At each visit, patients underwent comprehensive ophthalmological examinations, including the evaluation of best-corrected visual acuity (BCVA) (decimal), slit lamp examination, and dilated fundus examination.

Surgical procedure

The primary surgery involved pars plana vitrectomy using a 23-gauge vitrectomy system. Following fluid-air exchange, endolaser photocoagulation was performed, and SO was injected into the vitreous cavity. The same brand of SO (ophthafutur® sil 5000, Pharmpur GmbH, Königsbrunn, Germany) was used in all cases. After three to six months, SO was removed via the pars plana approach using a hybrid 25-gauge vitrectomy system with a 20-gauge sclerotomy for silicone Oil extrusion. The Stellaris Elite™, Bausch & Lomb, was employed for both procedures. All surgical procedures were carried out by the same retinal surgeon (C.Kour.) under retrobulbar anesthesia.

OCTA measurements

OCTA scans were performed using a spectral domain system (RTVue-XR Avanti, AngioVue, Optovue). All patients underwent HD Angio Retina 6.0 mm and HD Angio Disc 4.5 mm in both eyes. The scans were performed one day before the SO removal and then at 1 week and 1, 3, 6, and 12 months postoperatively. OCTA scans were repeated until good quality was obtained (image quality>5/10) to eliminate artifacts.

The main outcomes measured in this study regarding HD Angio Retina 6.0 mm scans included changes in (1) the foveal avascular zone (FAZ) area; (2) flow in the outer retina and choriocapillaris; (3) vessel density in the superficial and deep capillary plexus (total, superior-hemi, inferior-hemi, fovea, parafovea, perifovea); (4) retinal thickness (total, superior-hemi, inferior-hemi, fovea, parafovea, perifovea).

**Figure 1:**
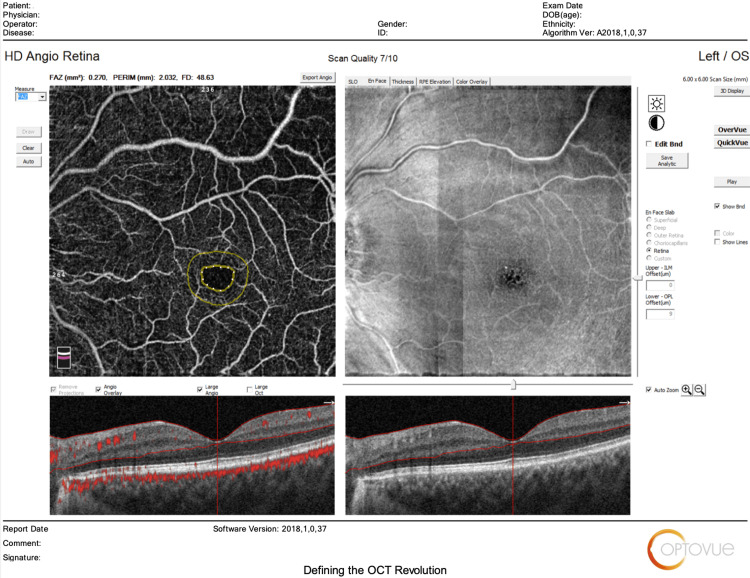
Optical coherence tomography angiography (OCTA) scan of HD Angio retina: Foveal avascular zone area.

**Figure 2:**
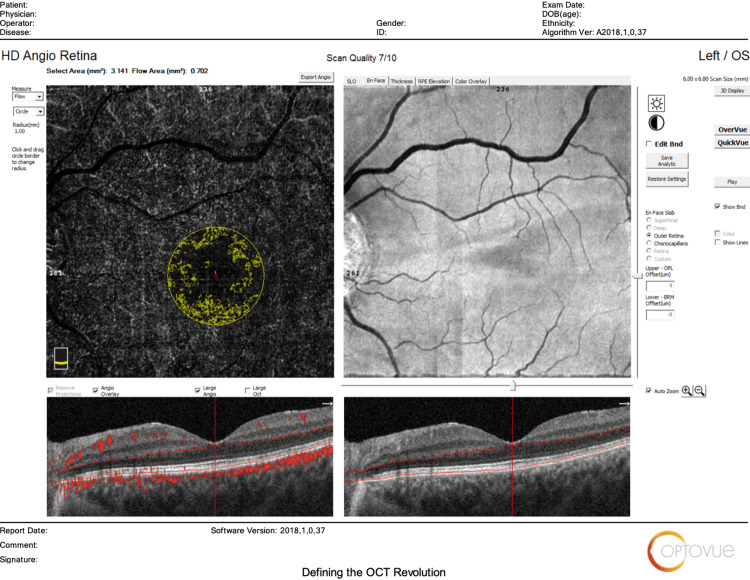
Optical coherence tomography angiography (OCTA) scan of HD Angio retina: flow in the outer retina.

**Figure 3:**
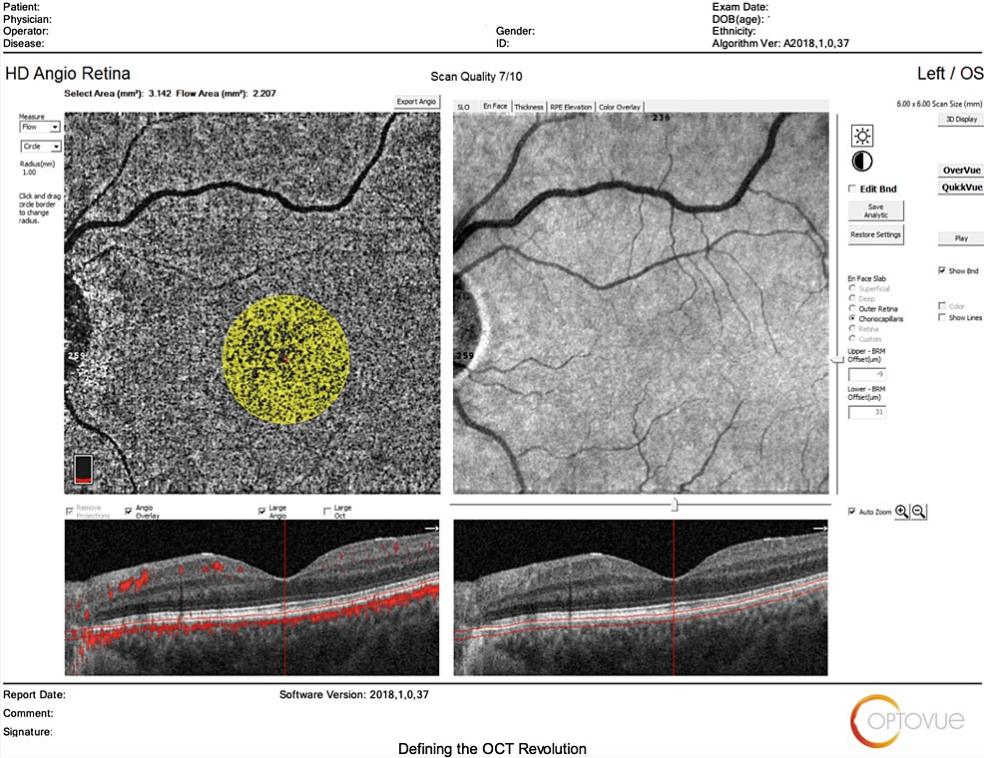
Optical coherence tomography angiography (OCTA) scan of HD Angio retina: flow in the choriocapillaris.

**Figure 4:**
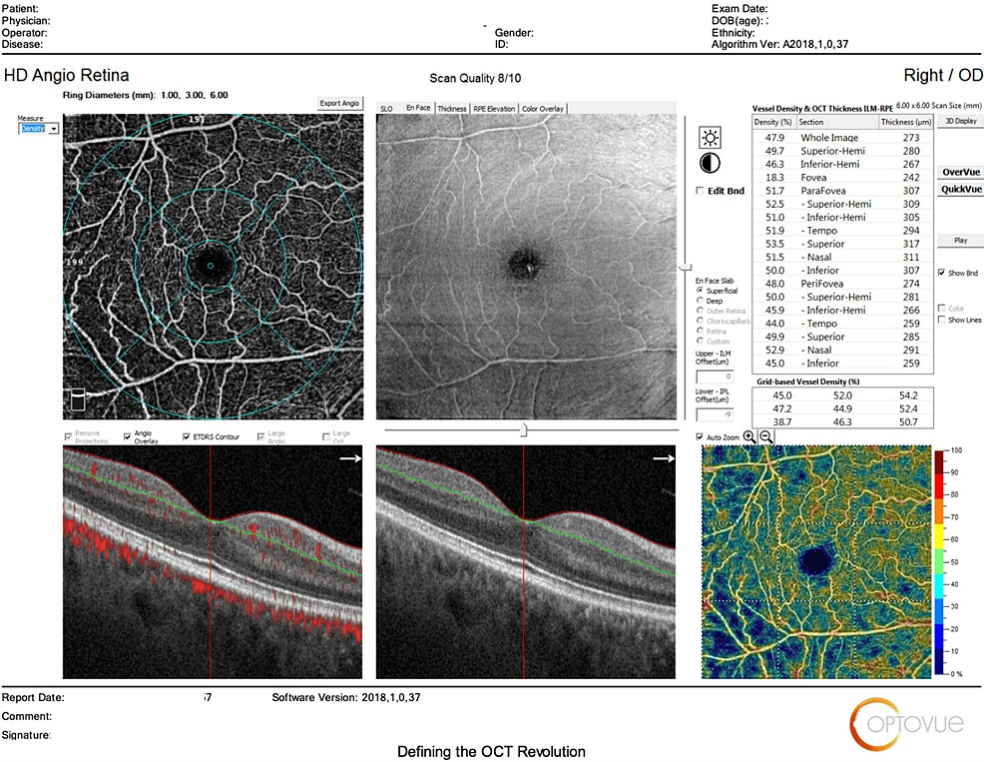
Optical coherence tomography angiography (OCTA) scan of HD Angio retina: vessel density in the superficial capillary plexus.

**Figure 5:**
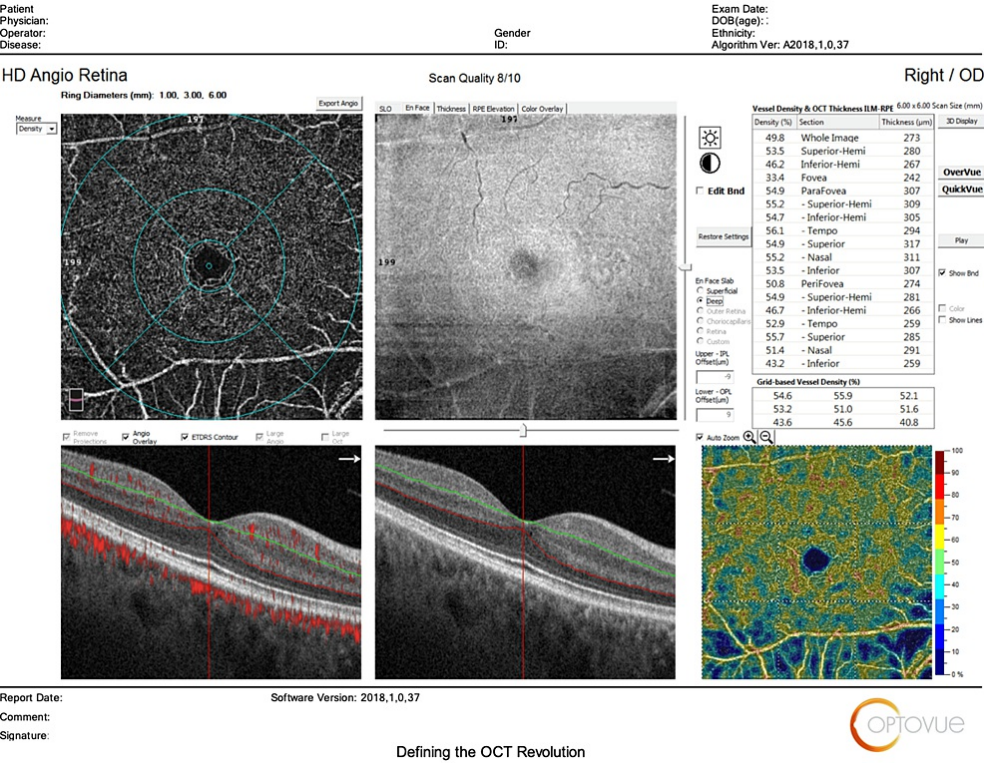
Optical coherence tomography angiography (OCTA) scan of HD Angio retina: vessel density in the deep capillary plexus.

**Figure 6:**
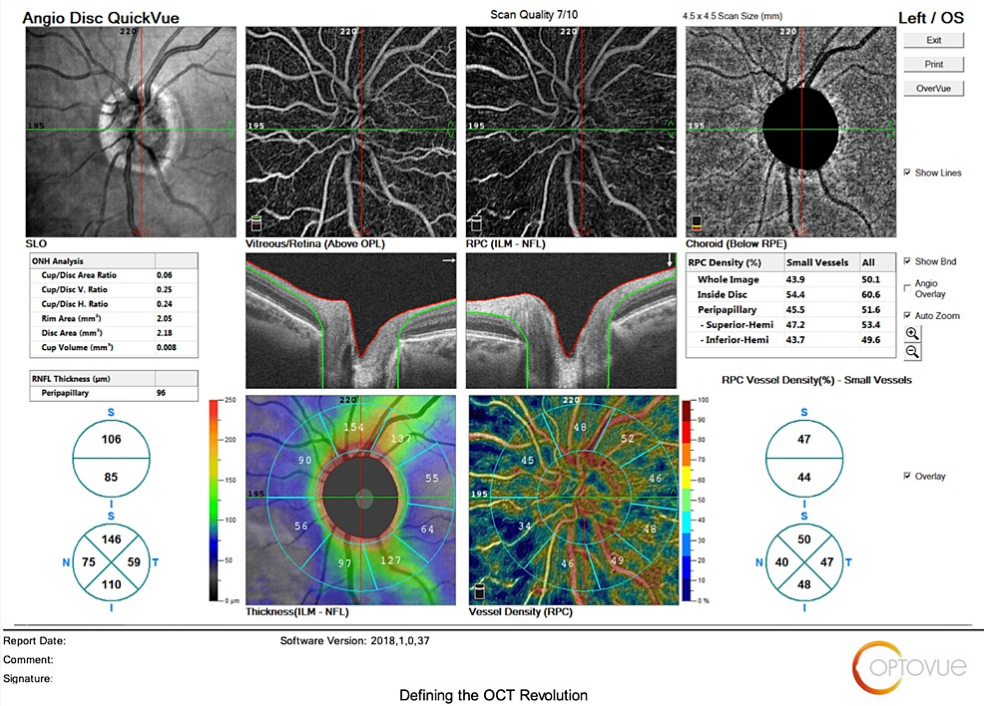
Optical coherence tomography angiography (OCTA) scan of HD Angio disc showing the optic nerve head analysis, the peripapillary retinal nerve fiber layer, and the radial peripapillary capillary (RPC) density for small vessels and all vessels.

The main outcomes measured regarding HD Angio Disc 4.5 mm scans included changes in (1) optic nerve head analysis (cup/disc area ratio, vertical and horizontal cup/disc ratio (CDVR and CDHR, respectively), rim area, disc area, and cup volume); (2) peripapillary retinal nerve fiber layer (RNFL) (total, superior, temporal, inferior, nasal); (3) radial peripapillary capillary (RPC) density for small vessels and all vessels (whole, inside disc, peripapillary(superior-hemi, inferior-hemi)). Figures 1-6 show example OCTA scans of HD Angio Retina and HD Angio Disc.

Statistical analysis

Basic characteristics of the patients were summarized using means and standard deviations (SD) for normally distributed continuous variables, and medians and interquartile ranges (IQRs) for skewed data. A comprehensive analysis was conducted on the operated eyes to evaluate changes in the aforementioned OCTA measurements over multiple time points. Analysis of Variance (ANOVA) tests were conducted for each measurement across predefined time points. Tukey's Honestly Significant Difference (HSD) test was performed for post hoc analysis in cases of significant ANOVA results. A series of independent t-tests were conducted to compare the aforementioned OCTA measurements between the intervention and control groups at different time points. ANOVA was also performed to investigate the relationship between BCVA and the OCTA measurements, as well as the change in BCVA over time.

A two-sided p-value of less than 0.05 was considered statistically significant. All analyses were carried out using the R programming language and the RStudio IDE. Patients’ health records were analyzed, and if missing values were detected, they were omitted [8,9].

## Results

A total of 28 patients were included in the study, comprising 18 males and 10 females. Two patients were excluded from the study due to loss of follow-up, and one patient was excluded due to low-quality scans resulting from poor ocular surface conditions. In the intervention group, 17 were right eyes, and 11 were left eyes. The mean age at the time of SO removal was 65 ± 10 years. The basic characteristics of the patients are summarized in Tables 1-8.

**Table 1:** Summary of Retina OCTA Parameters for All Six Time Points. OCTA: coherence tomography angiography; FAZ: Foveal avascular zone; SD: standard deviation; Time point 1: preoperatively; Time point 2: first postoperative week; Time point 3: first postoperative month; Time point 4: third postoperative month; Time point 5: sixth postoperative month; Time point 6: 12th postoperative month.

Group	Time Point	Patient Count	Mean FAZ±SD (mm^2^)	Mean Flow Area in Outer Retina±SD (mm^2^) (Selected Area = 3.142 mm^2^)	Mean Flow Area in Choriocapillaries±SD (mm^2^) (Selected Area = 3.142 mm^2^)	Mean Superficial Vessel Density±SD (%) (Whole Image)	Mean Superficial Vessel Density±SD (%) (Superior-Hemi)	Mean Superficial Vessel Density±SD (%) (Inferior-Hemi)
Intervention	1	28	0.220±0.135	1.075±0.546	2.050±0.133	41.3±5.7	41.2±5.5	41.3±5.9
	2	27	0.278±0.323	1.372±0.660	2.018±0.234	41.5±6.1	41.6±6.2	41.3±6.4
	3	28	0.283±0.290	1.118±0.728	2.081±0.109	41.8±4.7	53.0±5.6	41.6±5.6
	4	25	0.227±0.094	0.811±0.711	2.004±0.172	42.3±6.3	42.6±6.0	41.9±7.1
	5	23	0.211±0.097	0.722±0.517	2.033±0.134	42.8±5.6	43.4±5.9	42.1±5.7
	6	18	0.199±0.031	0.783±0.449	2.083±0.126	41.9±5.7	42.1±5.7	41.7±5.7
Control	1	27	0.221±0.121	0.812±0.474	2.081±0.166	46.7±7.0	46.6±6.7	46.8±7.7
	2	27	0.232±0.131	0.775±0.496	2.088±0.153	45.4±5.8	45.4±5.7	45.3±6.0
	3	26	0.226±0.104	0.740±0.450	2.115±0.146	46.3±4.6	46.3±4.8	46.2±4.8
	4	23	0.333±0.413	0.655±0.487	2.072±0.159	46.3±5.6	46.2±5.3	46.4±6.3
	5	22	0.232±0.151	0.846±0.597	2.043±0.208	47.0±7.8	47.4±7.7	47.5±9.0
	6	15	0.203±0.160	1.048±0.558	2.009±0.240	44.5±6.1	44.8±6.0	44.1±6.7

**Table 2:** Summary of Retina OCTA Parameters for All Six Time Points. OCTA: coherence tomography angiography; SD: standard deviation; Time point 1: preoperatively; Time point 2: first postoperative week; Time point 3: first postoperative month; Time point 4: third postoperative month; Time point 5: sixth postoperative month; Time point 6: 12th postoperative month.

Group	Time Point	Patient count	Mean Superficial Vessel Density±SD (%) (Fovea)	Mean Superficial Vessel Density±SD (%) (Parafovea)	Mean Superficial Vessel Density±SD (%) (Perifovea)	Mean Deep Vessel Density±SD (%) (Whole Image)	Mean Deep Vessel Density±SD (%) (Superior-Hemi)
Intervention	1	28	21.3±8.9	40.8±8.8	41.6±6.1	38.5±4.9	38.5±5.3
	2	27	20.0±8.2	41.8±8.3	42.1±6.3	38.3±6.8	38.1±6.7
	3	28	24.0±7.8	42.1±6.0	42.1±4.8	41.3±5.1	41.7±5.8
	4	25	22.3±8.6	42.1±8.0	42.6±6.6	41.8±7.1	42.3±7.9
	5	23	24.7±11.5	41.4±8.0	42.3±5.7	41.0±5.8	41.1±6.6
	6	18	26.1±9.4	40.9±5.2	42.5±6.6	42.9±4.7	43.7±5.7
Control	1	27	25.2±12.8	47.8±7.8	47.5±7.0	44.2±6.5	44.6±6.9
	2	27	24.0±8.6	46.6±7.4	46.3±5.6	44.5±7.5	45.5±7.6
	3	26	23.7±7.6	47.7±5.4	47.0±4.4	45.5±5.6	46.6±5.6
	4	23	24.5±8.2	47.5±9.6	47.2±4.9	46.1±6.8	46.5±6.2
	5	22	25.8±10.3	47.5±9.1	47.6±7.6	44.4±7.0	45.5±7.0
	6	15	29.7±12.4	45.5±7.4	44.8±5.8	43.2±7.1	43.5±7.4

**Table 3:** Summary of Retina OCTA Parameters for All Six Time Points. OCTA: coherence tomography angiography; SD: standard deviation; Time point 1: preoperatively; Time point 2: first postoperative week; Time point 3: first postoperative month; Time point 4: third postoperative month; Time point 5: sixth postoperative month; Time point 6: 12th postoperative month.

Group	Time Point	Patient Count	Mean Deep Vessel Density±SD (%) (Inferior-Hemi)	Mean Superficial Vessel Density±SD (%) (Fovea)	Mean Superficial Vessel Density±SD (%) (Parafovea)	Mean Superficial Vessel Density±SD (%) (Perifovea)	Mean Retinal Thickness±SD (μm) (Whole Image)
Intervention	1	28	38.1±5.8	31.6±10.2	44.9±6.3	38.2±5.5	286±30
	2	27	38.6±7.2	32.6±8.8	45.0±8.9	38.1±7.5	295±55
	3	28	41.0±5.1	37.1±6.5	48.4±5.0	41.7±5.9	291±38
	4	25	41.7±6.9	34.9±8.9	47.8±6.6	42.1±8.1	297±37
	5	23	40.9±5.7	33.3±7.3	46.8±6.3	41.8±7.0	299±41
	6	18	42.2±4.1	36.5±6.3	48.4±3.8	43.6±5.8	294±67
Control	27	28	43.7±6.4	37.3±12.0	48.7±5.1	45.0±7.0	282±24
	27	27	43.5±7.7	38.1±8.8	48.5±7.6	45.3±8.1	282±23
	26	28	44.5±5.9	37.3±7.8	49.7±5.4	46.2±6.4	278±26
	23	25	45.8±7.9	39.0±9.3	50.2±8.6	47.3±7.1	279±26
	22	23	43.4±7.4	37.1±9.5	49.2±6.8	45.8±7.7	282±28
	15	18	42.8±7.1	37.7±10.7	47.5±6.6	43.3±7.6	302±44

**Table 4:** Summary of Retina OCTA Parameters for All Six Time Points. OCTA: coherence tomography angiography; FAZ: Foveal avascular zone; SD: standard deviation; Time point 1: preoperatively; Time point 2: first postoperative week; Time point 3: first postoperative month; Time point 4: third postoperative month; Time point 5: sixth postoperative month; Time point 6: 12th postoperative month.

Group	Time Point	Patient Count	Mean Superficial Vessel Density±SD (%) (Fovea)	Mean Superficial Vessel Density±SD (%) (Parafovea)	Mean Superficial Vessel Density±SD (%) (Perifovea)	Mean Deep Vessel Density±SD (%) (Whole Image)	Mean Deep Vessel Density±SD (%) (Superior-Hemi)
Intervention	1	28	21.3±8.9	40.8±8.8	41.6±6.1	38.5±4.9	38.5±5.3
	2	27	20.0±8.2	41.8±8.3	42.1±6.3	38.3±6.8	38.1±6.7
	3	28	24.0±7.8	42.1±6.0	42.1±4.8	41.3±5.1	41.7±5.8
	4	25	22.3±8.6	42.1±8.0	42.6±6.6	41.8±7.1	42.3±7.9
	5	23	24.7±11.5	41.4±8.0	42.3±5.7	41.0±5.8	41.1±6.6
	6	18	26.1±9.4	40.9±5.2	42.5±6.6	42.9±4.7	43.7±5.7
Control	1	27	25.2±12.8	47.8±7.8	47.5±7.0	44.2±6.5	44.6±6.9
	2	27	24.0±8.6	46.6±7.4	46.3±5.6	44.5±7.5	45.5±7.6
	3	26	23.7±7.6	47.7±5.4	47.0±4.4	45.5±5.6	46.6±5.6
	4	23	24.5±8.2	47.5±9.6	47.2±4.9	46.1±6.8	46.5±6.2
	5	22	25.8±10.3	47.5±9.1	47.6±7.6	44.4±7.0	45.5±7.0
	6	15	29.7±12.4	45.5±7.4	44.8±5.8	43.2±7.1	43.5±7.4

**Table 5:** Summary of Optic Disc OCTA Parameters for All Six Time Points. OCTA: coherence tomography angiography; C/D: cup/disc ratio; SD: standard deviation; CDVR: cup/disc vertical ratio; CDHR: cup/disc horizontal ratio; Time point 1: Preoperatively; Time point 2: first postoperative week; Time point 3: first postoperative month; Time point 4: third postoperative month; Time point 5: sixth postoperative month; Time point 6: 12th postoperative month.

Group	Time Point	Patient Count	Mean C/D Area Ratio±SD	Mean CDVR±SD	Mean CDHR±SD	Mean Rim Area±SD (mm^2^)	Disc Area±SD (mm^2^)	Cup Volume±SD (mm^2^)
Intervention	1	28	0.11±0.14	0.27±0.28	0.21±0.21	1.74±0.39	1.97±0.36	0.03±0.05
	2	25	0.13±0.15	0.31±0.28	0.25±0.22	1.65±0.40	1.91±0.38	0.04±0.06
	3	28	0.12±0.13	0.29±0.27	0.23±0.20	1.60±0.28	1.81±0.29	0.03±0.05
	4	25	0.13±0.15	0.30±0.29	0.24±0.22	1.66±0.59	1.93±0.53	0.04±0.06
	5	22	0.13±0.14	0.30±0.29	0.23±0.22	1.50±0.41	1.81±0.33	0.09±0.24
	6	16	0.18±0.15	0.43±0.25	0.33±0.18	1.44±0.47	1.93±0.30	0.05±0.08
Control	1	25	0.10±0.13	0.25±0.25	0.21±0.20	1.78±0.39	1.98±0.40	0.03±0.05
	2	27	0.12±0.15	0.30±0.27	0.24±0.21	1.73±0.34	1.99±0.34	0.05±0.07
	3	25	0.11±0.13	0.26±0.26	0.22±0.21	1.72±0.34	1.95±0.37	0.04±0.07
	4	22	0.13±0.14	0.30±0.27	0.25±0.22	1.71±0.32	1.99±0.36	0.08±0.15
	5	21	0.11±0.13	0.26±0.26	0.22±0.21	1.75±0.35	2.00±0.44	0.04±0.06
	6	13	0.17±0.17	0.35±0.30	0.30±0.24	1.85±0.39	2.23±0.32	0.07±0.09

**Table 6:** Summary of Optic Disc OCTA Parameters for All Six Time Points. OCTA: coherence tomography angiography; RNFL: peripapillary retinal nerve fiber layer; Time point 1: preoperatively; Time point 2: first postoperative week; Time point 3: first postoperative month; Time point 4: third postoperative month; Time point 5: sixth postoperative month; Time point 6: 12th postoperative month.

Group	Time Point	Patient Count	Mean RNFL±SD (μm)	Mean Superior RNFL±SD (μm)	Mean Temporal RNFL±SD (μm)	Mean Inferior RNFL±SD (μm)	Mean Nasal RNFL±SD (μm)
Intervention	1	28	101±18	116±27	87±18	124±30	81±18
	2	25	106±21	123±29	87±20	130±31	89±26
	3	28	103±22	119±36	85±20	131±42	81±16
	4	25	103±33	124±45	90±36	126±40	79±24
	5	22	91±28	114±34	80±22	116±34	72±17
	6	16	90±20	112±33	75±17	113±33	68±14
Control	1	25	103±18	116±29	85±22	129±27	83±14
	2	27	102±17	116±29	84±23	128±24	84±15
	3	25	102±19	119±28	84±24	128±26	81±16
	4	22	102±18	116±29	84±24	130±26	83±14
	5	21	103±19	228±29	83±28	129±27	85±14
	6	13	105±11	122±21	83±15	135±21	87±15

**Table 7:** Summary of Optic Disc OCTA Parameters for All Six Time Points. OCTA: coherence tomography angiography; RPC: radial peripapillary capillary; Time point 1: preoperatively; Time point 2: first postoperative week; Time point 3: first postoperative month; Time point 4: third postoperative month; Time point 5: sixth postoperative month; Time point 6: 12th postoperative month.

Group	Time Point	Patient Count	Mean RPC Density of Small Vessels (Whole)±SD (%)	Mean RPC Density of Small Vessels (Inside disc)±SD (%)	Mean RPC Density of Small Vessels (Peripapillary)±SD (%)	Mean RPC Density of Small Vessels (Peripapillary Superior-Hemi)±SD (%)	Mean RPC Density of Small Vessels (Peripapillary Inferior-Hemi)±SD (%)
Intervention	1	28	41.8±4.9	44.8±6.5	43.3±6.3	42.3±7.4	43.7±7.2
	2	25	40.9±5.3	42.0±7.3	43.2±5.7	43.1±6.1	43.2±5.9
	3	28	41.8±5.0	44.5±7.8	43.5±6.3	43.2±7.4	43.8±6.1
	4	25	42.4±5.1	46.7±6.5	43.5±6.7	43.5±7.4	43.4±6.8
	5	22	43.4±6.3	47.6±7.2	44.1±7.3	43.0±6.5	42.5±7.2
	6	16	42.2±5.9	49.0±5.5	42.6±7.5	42.4±7.7	42.8±7.9
Control	1	25	46.9±4.8	50.5±5.3	49.2±6.3	48.5±7.4	49.9±5.4
	2	27	47.2±4.0	50.7±6.7	49.5±5.0	49.2±6.3	49.8±4.2
	3	25	46.8±5.2	50.3±6.8	48.8±6.7	48.7±7.9	49.0±5.8
	4	22	47.2±4.4	51.4±6.4	49.3±5.9	48.9±6.9	50.0±5.2
	5	21	46.9±4.7	50.3±8.2	49.2±6.4	49.1±8.0	49.3±5.4
	6	13	48.2±2.3	51.0±7.5	50.4±6.7	50.2±6.7	50.5±6.7

**Table 8:** Summary of Optic Disc OCTA Parameters for All Six Time Points. OCTA: coherence tomography angiography; RPC: radial peripapillary capillary; Time point 1: preoperatively; Time point 2: first postoperative week; Time point 3: first postoperative month; Time point 4: third postoperative month; Time point 5: sixth postoperative month; Time point 6: 12th postoperative month.

Group	Time Point	Patient Count	Mean RPC Density of All Vessels (Whole)±SD (%)	Mean RPC Density of All Vessels (Inside Disc)±SD (%)	Mean RPC Density of All Vessels (Peripapillary)±SD (%)	Mean RPC Density of All Vessels (Peripapillary Superior-Hemi)±SD (%)	Mean RPC Density of All Vessels (Peripapillary Inferior-Hemi)±SD (%)
Intervention	1	28	47.4±4.7	53.7±5.1	48.5±6.2	47.8±6.9	49.1±6.0
	2	25	46.5±4.7	50.9±6.0	48.2±5.2	48.3±5.6	48,1±5.5
	3	28	47.8±5.1	54.2±6.1	49.2±6.1	49.2±7.0	49.2±5.7
	4	25	48.5±5.2	55.4±5.3	49.2±6.4	49.5±7.4	49.1±6.2
	5	22	48.2±5.3	56.1±6.5	50.1±6.8	49.0±6.2	48.3±6.2
	6	16	48.0±5.6	57.0±3.9	48.6±7.3	48.5±7.3	48.5±7.6
Control	1	25	52.8±4.9	59.1±3.9	54.9±6.4	54.6±7.1	55.1±5.7
	2	27	53.1±4.2	59.0±5.4	55.2±5.3	55.2±6.4	55.1±4.5
	3	25	53.1±5.9	59.0±5.9	54.5±6.8	54.4±8.0	54.3±6.2
	4	22	53.2±4.5	59.4±5.3	55.1±5.9	55.1±6.6	55.2±5.8
	5	21	52.6±4.9	58.7±6.6	54.7±6.7	54.9±7.7	48.3±6.7
	6	13	54.2±4.1	59.4±5.7	56.2±6.1	56.4±6.0	56.1±6.2

Retina OCTA results

The ANOVA tests for the intervention group revealed no statistically significant changes over time for the following measurements: FAZ area, flow in the choriocapillaris, vessel density in the superficial capillary plexus (total, superior-hemi, inferior-hemi, fovea, parafovea, perifovea), vessel density in the deep capillary plexus (inferior-hemi, fovea, parafovea), and retinal thickness (total, superior-hemi, inferior-hemi, fovea, parafovea, perifovea). However, significant differences were observed in the flow of the outer retina (p = 0.0023) and vessel density in the deep capillary plexus (perifovea) (p = 0.0429).

Subsequent post-hoc analysis with Tukey's HSD test for these measurements was performed to identify specific time points where significant differences occurred.

**Figure 7:**
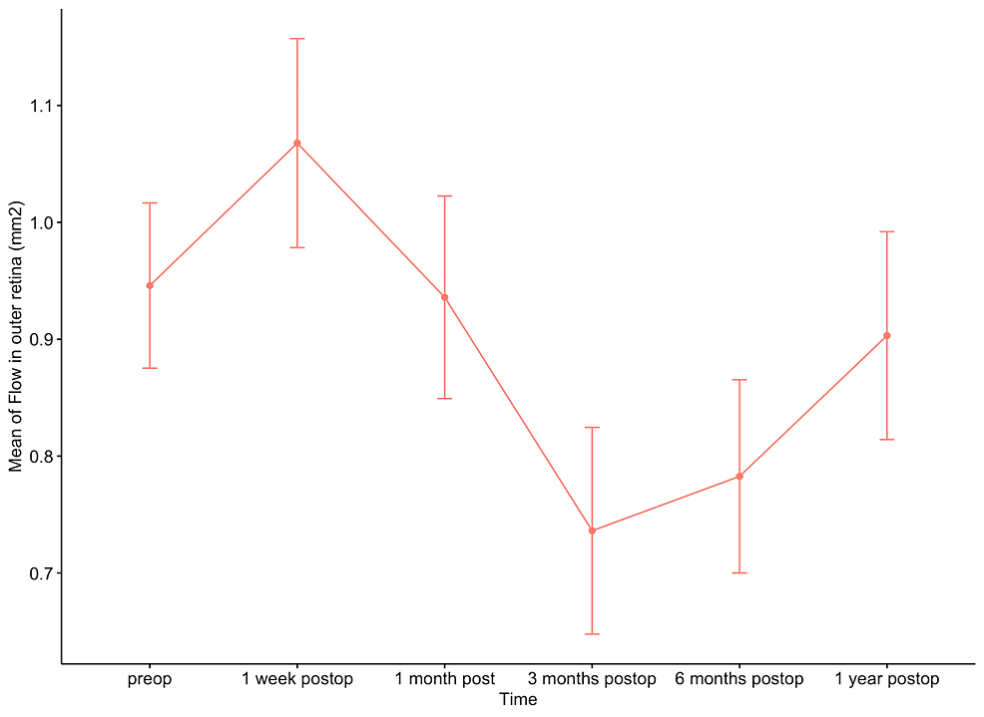
Means of flow in the outer retina during the observation period in the intervention group.

In the case of flow in the outer retina, significant mean differences were observed between the first postoperative week and the third postoperative month (mean difference = -0.561 mm², p = 0.02), between the first postoperative week and the sixth (mean difference = -0.650 mm², p = 0.005), and between the 1st postoperative week and the 12th postoperative month (mean difference = -0.600 mm², p = 0.03) (Figure 7).

In the case of vessel density in the deep capillary plexus (perifovea), while the ANOVA was significant, Tukey's HSD test did not reveal any specific pairwise time comparisons as significant.

A series of independent t-tests were conducted to compare retina OCTA measurements between the intervention and control groups at different time points.

No significant differences between the intervention and control group were observed in the following measurements: FAZ, flow in the outer retina and choriocapillaris, vessel density in the superficial capillary plexus (superior-hemi), and retinal thickness (total, inferior-hemi, fovea, parafovea).

**Figure 8:**
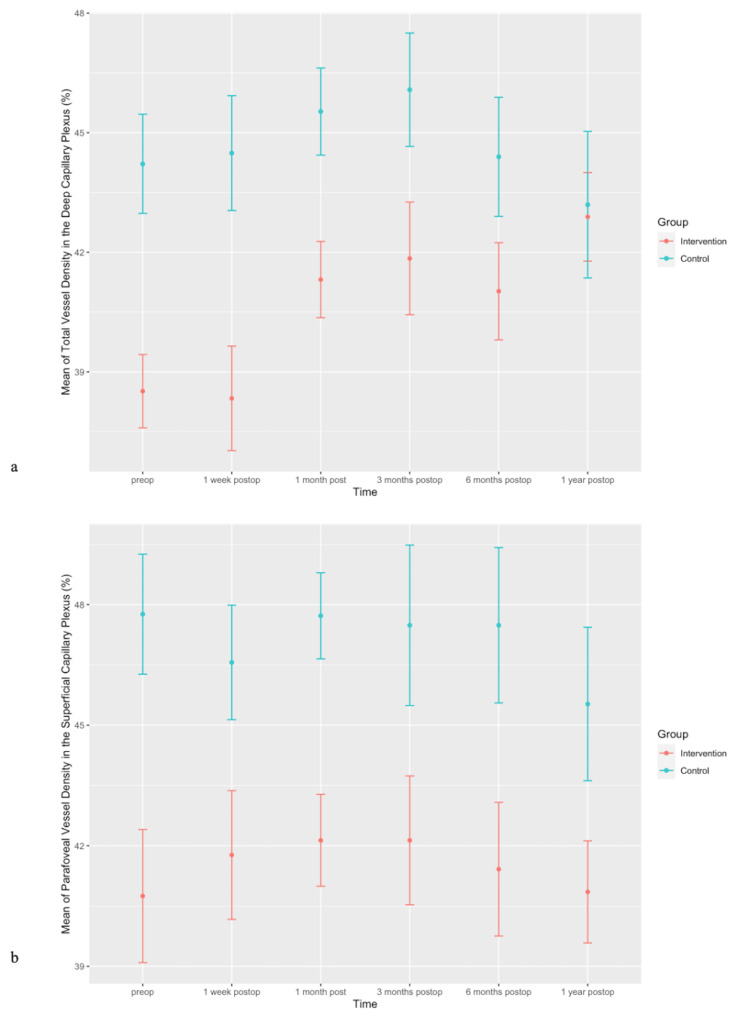
(a) Error bars of means of total vessel density in deep capillary plexus (%) during the observation period in both intervention and control groups (preop, p<0.001; one week postop, p<0.01; one month postop, p<0.01; three months postop, p<0.05; six months postop, p<0.05; one year postop, p<0.01). (b) Error bars of means of parafoveal vessel density in superficial capillary plexus (%) during the observation period in both the intervention and control groups (preop, p=0.01; one week postop, p=0.05; one month postop, p=0.123; three months postop, p<0.05; six months postop, p<0.05; one year postop, p<0.05).

Statistically significant differences between the intervention and control group were observed in vessel density in the superficial capillary plexus (total, inferior-hemi, fovea, parafovea, perifovea), vessel density in the deep capillary plexus (total, superior-hemi, inferior-hemi, fovea, parafovea, perifovea), and retinal thickness (superior-hemi) during the observation period (Figure 8).

Optic disc OCTA results

The ANOVA tests for the intervention group revealed no statistically significant changes over time for the following measurements: C/D area ratio, CDVR, CDHR, rim area, disc area, cup volume, RNFL (total, superior, temporal, inferior), and RPC density for small vessels and all vessels (whole, peripapillary - superior and inferior hemi).

However, RPC density for small and all vessels inside the disc, together with nasal RNFL, revealed significant ANOVA results, leading to further analysis using Tukey's HSD test.

Significant differences in nasal RNFL were observed between the first postoperative week and six postoperative months (mean difference = -17 μm, p=0.042), and between the first postoperative week and 12 postoperative months (mean difference = -21 μm, p=0.0163).

**Figure 9:**
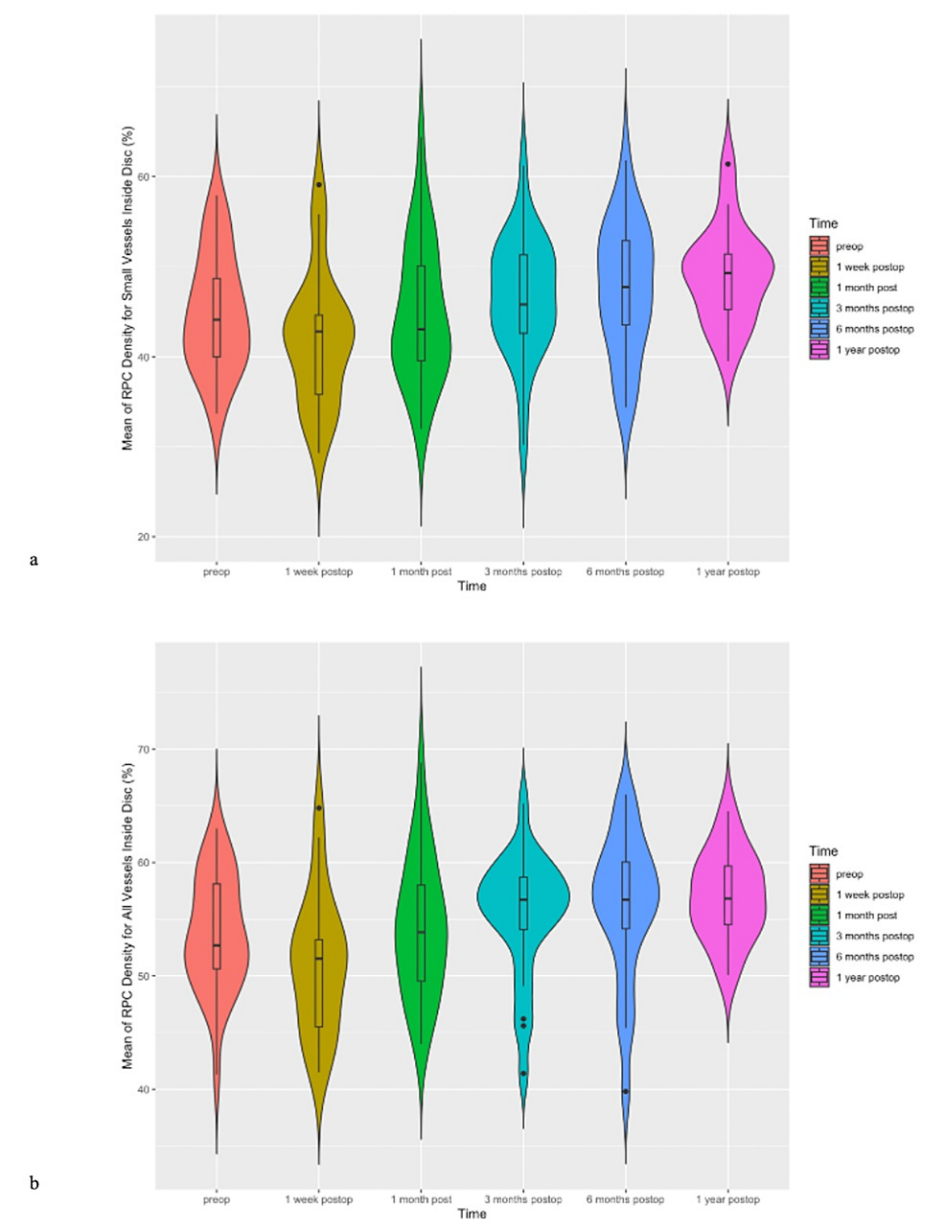
(a) Violin plots of means of radial peripapillary capillary (RPC) density for small vessels inside the disc during the observation period in the intervention group. (b) Violin plots of means of radial peripapillary capillary (RPC) density for all vessels inside the disc during the observation period in the intervention group.

Significant differences in RPC density for small vessels inside the disc were observed between the first postoperative week and 12 postoperative months (mean difference = 7.1%, p=0.021) (Figure 9).

Significant differences in RPC density for all vessels inside the disc were observed between the first postoperative week and six postoperative months (mean difference = 5.1%, p=0.0263) and between the first postoperative week and 12 postoperative months (mean difference = 7.1%, p=0.021) (Figure 9).

A series of independent t-tests were conducted to compare optic disc OCTA measurements between the intervention and control groups at different time points.

No significant differences between the intervention and control group were observed in the following measurements: C/D area ratio, CDVR, CDHR, rim area, disc area, cup volume, and RNFL (total, superior, temporal, inferior) (p>0.05).

**Figure 10:**
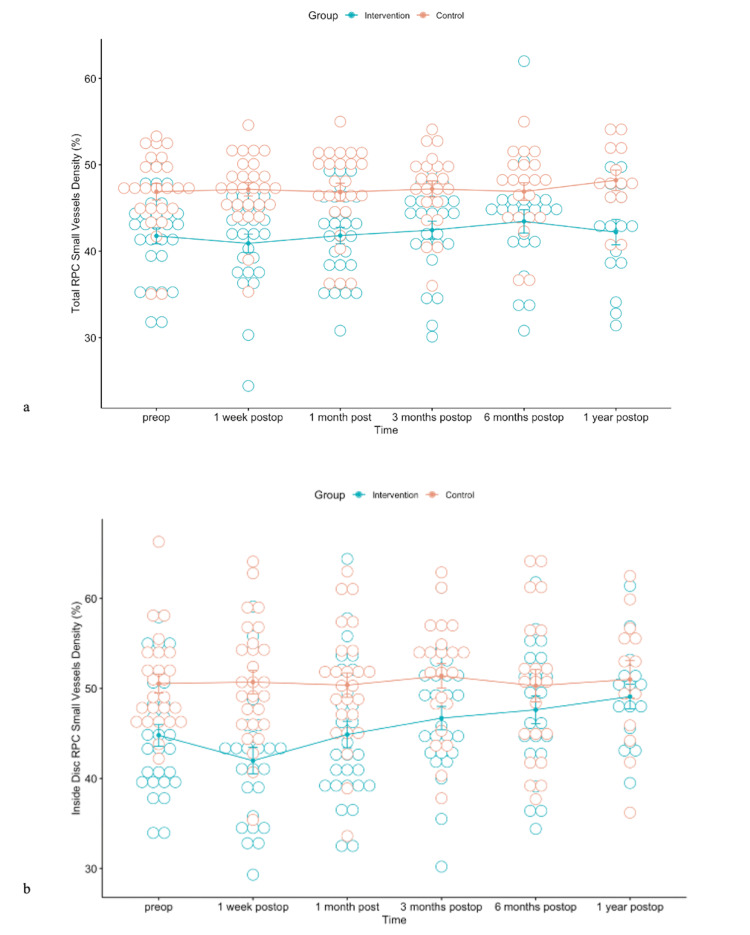
FIGURE 10: (a) Error bars for means of total radial peripapillary capillary (RPC) density for small vessels during the observation period for both intervention and control groups (preop, p<0.001; one week postop, p<0.0001; one month postop, p<0.001; three months postop, p<0.05, 6 months postop p<0.05, 1-year postop p<0.01). (b) Error bars for means of RPC density for small vessels inside the disc during the observation period in both the intervention and control groups (preop, p<0.001; one week postop, p<0.0001; one month postop, p<0.001; three months postop, p<0.05; six months postop, p=0.265; one year postop, p=0.434).

**Figure 11:**
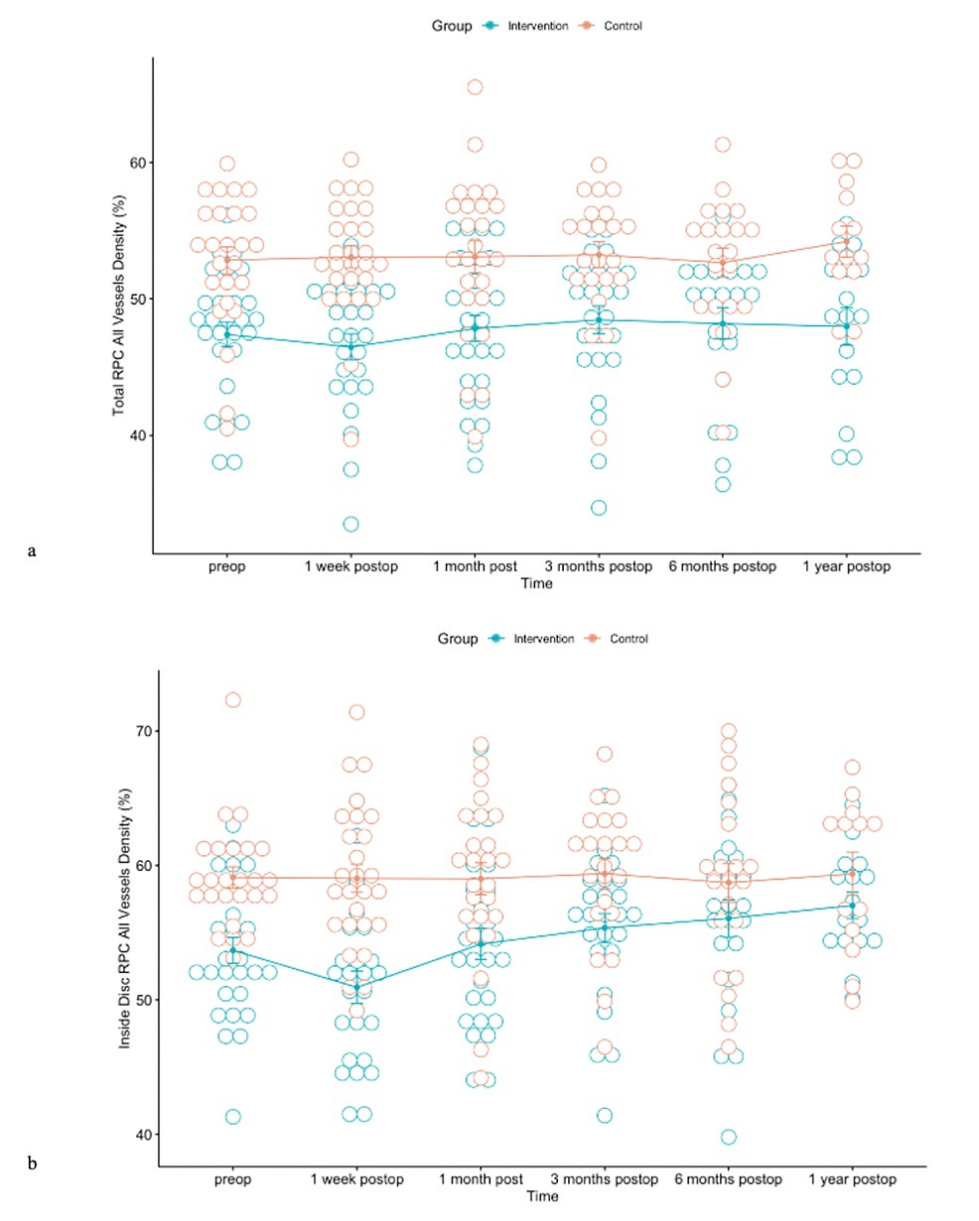
(a) Error bars for means of total RPC density for all vessels during the observation period for both intervention and control groups. (b) Error bars for means of RPC density for all vessels inside the disc during the observation period in both intervention and control groups.

Statistically significant differences between the intervention and control group were observed in RPC density for small and all vessels (whole, inside disc, peripapillary, peripapillary superior-hemi, peripapillary inferior-hemi) during the observation period (Figures 10, 11).

BCVA results

ANOVA was performed to investigate the relationship between BCVA and retina OCTA parameters, between BCVA and optic disc OCTA parameters, as well as the change in BCVA over time, in the intervention group.

There was a statistically significant association between BCVA and the following retina OCTA parameters: vessel density in the superficial capillary plexus [total (p<0.001), inferior-hemi (p<0.00001), parafovea (p<0.01), perifovea (p<0.001)].

There was a statistically significant association between BCVA and the following optic disc OCTA parameters: C/D area ratio (p=0.0336), CDVR (p=0.0484), nasal RNFL (p=0.016), RPC of small vessels density (whole (p=0.0018), inside disc (p<0.001), peripapillary (p=0.0114), peripapillary inferior hemi (p<0.001)], and RPC of all vessels density [whole (p=0.0017), inside disc (p<0.001), peripapillary (p<0.01), and peripapillary inferior hemi (p<0.001)).

**Figure 12:**
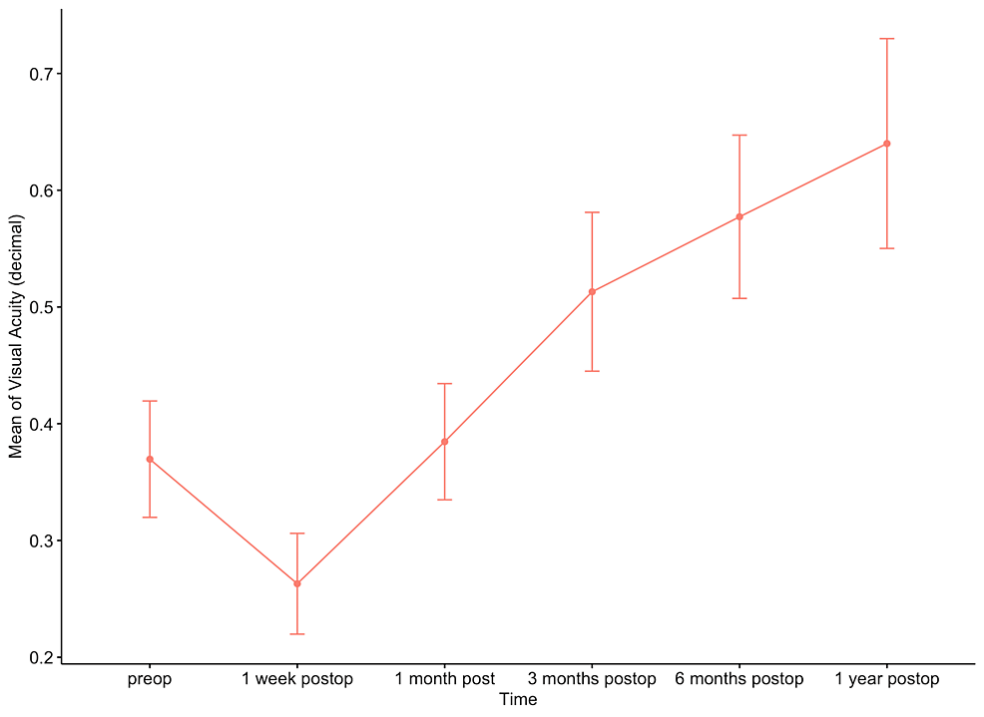
Means of best corrected visual acuity (decimal) during the observation period in the intervention group.

In addition, BCVA changed significantly over the observed time points (p<0.001) (mean differences (decimal) preop: 0.4 ±0.3, one week postop: 0.25±0.2, one month postop: 0.40±0.3, three months postop: 0.50±0.3, six months postop: 0.58±0.32, one year postop: 0.63±0.4) (Figure 12).

## Discussion

Significant changes in flow in the outer retina were observed between the first postoperative week and the third, sixth, and 12th postoperative months in the intervention group. Statistically significant differences between the intervention and control group were observed in vessel density in the superficial (parafovea) and deep (total) capillary plexus, and those changes remained significant until the first postoperative year.

Significant changes in RPC density for small and all vessels inside the disc were observed between the first postoperative week and 12 postoperative months in the intervention group.

The significant differences between the intervention and control groups, which were observed in total RPC density for small and all vessels, remained significant until the first postoperative year. In contrast, the significant differences between the intervention and control group, which were observed in RPC density for small and all vessels inside the disc, were significant only until the third postoperative month.

Previous studies have focused on the effects of SO tamponade on the retina. In their study, Lee et al. included 30 patients who underwent pars plana vitrectomy for RD with SO tamponade, and OCTA scans were performed before RD surgery, during SO tamponade, and after SO removal [10]. They showed that similarly to our results, six months after SO removal, the parafoveal thickness was not significantly different compared to that of fellow control eyes. Nevertheless, the peripapillary RNFL thickness was significantly decreased during SO tamponade and six months after SO removal, unlike our results, where changes solely in nasal RNFL were observed [10]. However, there were no significant differences in the FAZ and macular vessel density on the OCTA [10]. Another study included 38 patients with unilateral RRD treated with vitrectomy and SO tamponade [11]. The patients were followed up for more than 3 months after SO removal, using OCTA, and both FAZ and macular vessel density were evaluated by comparing the operated eyes with the unaffected contralateral eyes [11]. The authors observed that FAZ was larger and, similarly to the results of our study, the macular vessel density in the deep capillary plexus was lower in the operated eyes than in the fellow eyes. They significantly correlated those changes with the duration of SO tamponade and supposed that those changes may justify the unexplained visual loss [11].

Hou et al. investigated vascular changes in the macular and peripapillary regions before and after SO removal in patients with RD using OCTA [12]. They observed that macular superficial vessel density and superficial perfusion density decreased during SO tamponade and increased after SO removal [12]. In our study, the flow in the outer retina decreased until the 3rd postoperative month, after which, it increased again until the 1st postoperative year, without reaching the preoperative levels.

Regarding BCVA, a reduction was observed in the first postoperative week, but it had reached pre-operative levels by the 1st postoperative month, and it improved significantly until one year postoperatively. In addition, there was a statistically significant association between BCVA and vessel density in the superficial capillary plexus, C/D area ratio, and RPC density of small and all vessels. It should be highlighted that, based on previous studies, independent factors that may affect the final BCVA after RD include the age of the patients, the duration of the retinal detachment, and the pre-operative BCVA at the time of RD [13-16]. Previous studies have reported that unexplained visual loss may occur after SO removal, with an incidence of 3-13% [17-20]. This phenomenon has been associated with higher IOP and longer SO tamponade duration [17,21]. An interpretation of this phenomenon includes changes in blood perfusion to the retina at the time of SO removal and sudden dramatic physiochemical disturbance in the aqueous consistency, including potassium, which in turn results in reduced regulation of K+ levels and eventual neuronal cell damage [17,22]. However, in our study, unexplained visual loss did not occur in any patient, but this may be explained by the sample size of the study.

The results of this study should be interpreted within the context of its limitations. The observation period was initially set at one year, but 23 patients completed the six-month follow-up and 18 patients completed the one-year follow-up. The extended follow-up of more patients would further support the long-term results of the present study.

## Conclusions

SO removal seems to cause changes in macular vessel density, as well as in radial peripapillary capillary density, which seems to improve after the first postoperative month until the first postoperative year. In particular, those changes were observed between the first postoperative week and 6 and 12 postoperative months (p=0.0263 and p=0.021, respectively). BCVA is likely associated with those parameters, but improvement might be observed even one year following SO removal. Further studies are required to investigate other factors that may affect the BCVA outcome.
